# High-Performance Telescope System Design for Space-Based Gravitational Waves Detection

**DOI:** 10.3390/s24227309

**Published:** 2024-11-15

**Authors:** Huiru Ji, Lujia Zhao, Zichao Fan, Rundong Fan, Jiamin Cao, Yan Mo, Hao Tan, Zhiyu Jiang, Donglin Ma

**Affiliations:** 1 MOE Key Laboratory of Fundamental Physical Quantities Measurement and Hubei Key Laboratory of Gravitation and Quantum Physics, PGMF and School of Physics, Huazhong University of Science and Technology, Wuhan 430074, China; huiruji@hust.edu.cn (H.J.); fanzichao@hust.edu.cn (Z.F.); caojiamin@hust.edu.cn (J.C.); ammo0925@hust.edu.cn (Y.M.); tanhao960410@hust.edu.cn (H.T.); jiangzy@hust.edu.cn (Z.J.); 2 School of Optical and Electronic Information and Wuhan National Laboratory of Optoelectronics, Huazhong University of Science and Technology, Wuhan 430074, China; frd0823@hust.edu.cn; 3 Shenzhen Huazhong University of Science and Technology, Shenzhen 518057, China

**Keywords:** gravitational waves detection, spaceborne telescope, optical system design

## Abstract

Space-based gravitational wave (GW) detection employs the Michelson interferometry principle to construct ultra-long baseline laser interferometers in space for detecting GW signals with a frequency band of 10^−4^–1 Hz. The spaceborne telescope, as a core component directly integrated into the laser link, comes in various configurations, with the off-axis four-mirror design being the most prevalent. In this paper, we present a high-performance design based on this configuration, which exhibits a stable structure, ultra-low wavefront aberration, and high-level stray light suppression capabilities, effectively eliminating background noise. Also, a scientifically justified positioning of the entrance and exit pupils has been implemented, thereby paving adequate spatial provision for the integration of subsequent optical systems. The final design realizes a wavefront error of less than λ/500 in the science field of view, and after tolerance allocation and Monte Carlo analysis, a wavefront error of less than λ/30 can be achieved with a probability of 92%. The chief ray spot diagram dimensions are significantly small, indicating excellent control of pupil aberrations. Additionally, the tilt-to-length (TTL) noise and stray light meet the stringent requirements for space-based gravitational wave detection. The refined design presented in this paper proves to be a more fitting candidate for GW detection projects, offering more accurate and rational guidance.

## 1. Introduction

In 2015, the ground-based gravitational wave (GW) detector LIGO announced the first direct detection of GWs (GW150914 event) [1]. The essence of GW is the change in the space-time structure caused by the asymmetric acceleration of two supermassive objects. Ground-based laser interferometric gravitational wave detection systems typically feature L-shaped measurement arms with lengths on the order of kilometers. Besides LIGO, notable examples include VIRGO [2], GEO600 [3], and TAMA300 [4]. However, ground-based detectors are limited by surface vibrations, lunar gradient noise, and interferometer arm length, restricting their detection capabilities to frequencies above 1 Hz [5,6,7,8]. To achieve higher sensitivity of GW signal detection, space-based GW detection projects such as The Laser Interferometer Space Antenna (LISA) [9], TianQin [10], and Taiji [11] have been proposed, which can detect signals with a frequency band of 10^−4^–1 Hz.

Space-based GW detection utilizes the Michelson interferometry principle to construct ultra-long baseline laser interferometers. These detectors accurately measure the displacement changes between test masses placed at the ends of the ultra-long baseline to detect GW signals. Given the extremely weak nature of GW signals, take the GW150914 event as an example, where the relative strength was about 10^−21^. Using space-based GW detection, even with interferometer arm lengths of tens or hundreds of kilometers, the displacement changes induced by GWs are still only on the picometer scale. Accurately measuring such minute distance changes over such long baselines imposes stringent requirements on the optical path stability of the space-based interferometric system.

The space-based GW detection telescope is directly integrated into the optical path of the interferometer, serving as a core component of the space GW detector. Its primary functions are to switch beam sizes and facilitate beam transmission between spacecraft. These functions require the telescope to simultaneously emit and receive beams from distant spacecraft. At the receiving end, the telescope reduces a large-aperture beam to a small-aperture collimated beam, while at the emitting end, it expands a small-aperture beam to a near-diffraction-limited large-aperture collimated beam. Due to the extremely weak signals and inherent surface roughness from manufacturing, the GW detection telescope must exhibit high optical path stability and low wavefront aberration. Additionally, high-energy lasers inevitably produce backscatter, generating additional phase noise. The telescope structure needs to suppress stray light to ensure precise detection of the signal light.

Therefore, when selecting the configuration for the GW detection telescope, the above factors should be fully considered. Off-axis reflective systems, due to their lack of central obstruction, high magnification capability, and excellent stray light suppression, have become the mainstream choice. As a collaborative mission led by the European Space Agency (ESA) and the National Aeronautics and Space Administration (NASA), LISA was initially proposed in the 1990s [9,12,13]. It involves three spacecraft forming an equilateral triangle with sides approximately 2.5 million kilometers, orbiting the Sun following the Earth. These spacecrafts use space-borne telescopes to send and receive laser beams between each other, establishing a laser link to precisely measure the distance variations between test masses. The design of spaceborne telescopes has undergone multiple improvements, evolving from coaxial systems to off-axis six-mirror and off-axis four-mirror configurations [14]. In the latest LISA studies, Livas et al. introduced freeform surfaces in the off-axis four-mirror configuration to correct pupil aberrations and reduce optical path coupling noise [15]. Luo et al. proposed the TianQin project, which involves deploying three microsatellites in an equilateral triangle formation with 170,000 km arms at an altitude of 100,000 km and is based on a geocentric orbital configuration [10]. In terms of telescope configuration, Fan et al. presented an off-axis four-mirror spaceborne telescope design, achieving ultra-low wavefront distortion for beam transmission [16,17]. Building upon these works, this design still has many areas for improvement, which will be discussed in detail in this paper. Another Chinese space-based GW detection project, the Taiji program, has a design concept similar to LISA. Wang et al. provided a preliminary design for a prototype with a 200 mm aperture [18,19].

This paper consists of seven sections. Section 2 lists and analyses the optical design parameters along with their rationale. The design methodologies are introduced in Section 3, including the calculation of initial structural aberrations and the construction of the optimization merit function. Section 4 presents the specific initial design parameters and the obtained final design results. Then, in Section 5, the performance of the final system from three aspects is analyzed. In Section 6, the feasibility of assembly and manufacturing is evaluated through tolerance analysis of the system, while a simple stray light analysis results and a thermal analysis are addressed. The concluding section summarizes the main content of the entire paper.

## 2. Design Specifications

The design specifications of the telescope are outlined in Table 1, which references the requirements for the TianQin project [16,17,20] and other space-based GW missions [15,19,21,22,23]. The spatial GW detection telescope operates at a wavelength of 1064 nm, determined by the mature and reliable ND: YAG solid-state laser used in the scientific interferometer [16]. This wavelength helps reduce energy loss from diffraction during ultra-long-range transmission. Throughout the scientific operation of GW detection projects, the laser must consistently provide stable output power with high frequency and phase stability.

To reduce the level of backscattering, the optical system of the space GW detection telescope still uses an unobstructed off-axis four-mirror optical system. All-reflective systems can be unobscured to maximize the light throughput for a given aperture size. The primary mirror (PM) and secondary mirror (SM) focus the parallel input beam onto the intermediate image plane, while the tertiary mirror (TM) and quaternary mirror (QM) realign the beam into a parallel beam with low wavefront error. Generally, a stop is placed at the intermediate image surface to reduce stray light effectively. Additionally, as the telescope needs to match the local device, the telescope has a real exit pupil. The definition of the entrance and exit pupils will affect the analysis of the system’s pupil aberrations. Compared to the previous work [16,17], this paper designs with reference to the layout of the LISA telescope [14,15], particularly making a more reasonable arrangement of the positions of the entrance and exit pupils. The entrance pupil position has been shifted to 300 mm in front of PM, with a constraint ensuring the exit pupil remains at least 200 mm behind QM, which is more reasonable for the receiving and output distances of the light beam.

This study places particular emphasis on discerning wavefront aberration issues corresponding to the acquisition Field-of-Regard (FOR) and the science Field-of-View (FOV). The former’s viewing corners are relatively large, and the purpose is to quickly realize the construction of space laser interference links, while the range of the science FOV is relatively small to achieve stable laser transmission. According to the overall index distribution, this telescope has an acquisition FOR of 400 μrad and a science FOV of 14 μrad.

The wavefront quality of the telescope will affect the energy transmission efficiency of the laser interferometer system and the input of the tilt-to-length (TTL) noise [20,24]. Superior wavefront quality is a necessary condition for achieving high-efficiency energy transmission. Asymmetric wavefront errors will significantly affect TTL noise. To minimize TTL noise, the telescope system’s wavefront transmission should be close to the diffraction limit and present as a low-distortion plane wave. In order to reduce TTL noise as much as possible and leave margin for assembly and manufacturing errors, the design residual wavefront error should not be greater than λ/300. The optical path stability of a telescope is related to optical path noise and pointing accuracy. Optical path stability on the picometer scale requires a TTL noise level of approximately 0.025 nm/μrad. The stray light budget of the mirror is related to the signal light power, and the stray light power needs to be less than 10^−10^ of the laser source power.

## 3. Structural Design and Optimization Methods

### 3.1. Initial System Design with Aberration Calculation

The design of the GW detection telescope is based on an off-axis four-mirror configuration. In the preliminary stages, the first two mirrors and the last two mirrors can be considered as two separate imaging telescopes. PM and SM typically employ an RC (Ritchey–Chrétien) configuration, which is modified for off-axis use to eliminate obscuration. The design of this paper is based on Seidel aberration theory for aberration calculation of coaxial two-mirror initial structure. Initially, only conic and spherical surfaces are used, avoiding the use of aspherical and other higher-order surface types.

**Figure 1:**
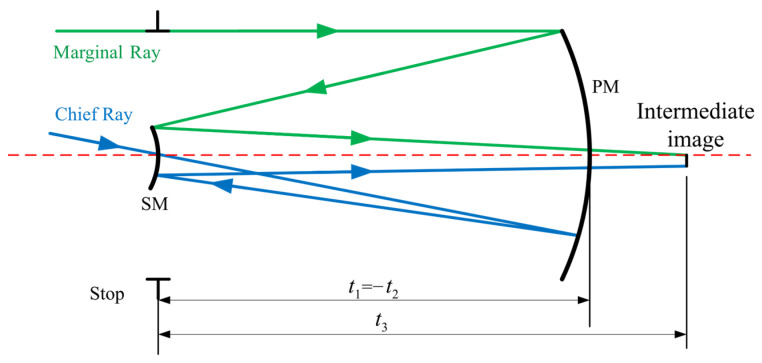
Raytracing of the initial coaxial PM and SM.

As illustrated in Figure 1, $t_{1}$ and $t_{2}$ represent the distance between the entrance pupil and PM, the distance between PM and SM, which are both set to 300 mm and kept at approximately the same position in subsequent designs to facilitate assembly and adjustment. For this coaxial two-mirror system, characteristic ray tracing is conducted by tracing both a chief ray and a marginal ray.

According to Seidel aberration theory, we can get the corresponding aberration coefficients as shown in Equation (1).

(1) $$\left\{\begin{array}{l} S_{I}=S_{I}^{s}+\delta S_{I}^{a}\\ S_{\mathrm{II}}=S_{\mathrm{II}}^{s}+\delta S_{\mathrm{II}}^{a}\\ S_{\mathrm{III}}=S_{\mathrm{III}}^{s}+\delta S_{\mathrm{III}}^{a}\\ S_{\mathrm{IV}}=S_{\mathrm{IV}}^{s}\\ S_{V}=S_{V}^{s}+\delta S_{V}^{a} \end{array}\right..$$

where S1 to S5 separately represent the system’s total spherical aberration ($S_{I}$), coma ($S_{\mathrm{II}}$), astigmatism ($S_{\mathrm{III}}$), field curvature ($S_{\mathrm{IV}}$), and distortion ($S_{V}$). Each coefficient contains a spherical component ($S_{I}^{s}$~$S_{V}^{s}$) and an aspherical component ($\delta S_{I}^{a}$, $\delta S_{\mathrm{II}}^{a}$, $\delta S_{\mathrm{III}}^{a}$, and $\delta S_{V}^{a}$). Due to space limitations, the specific derivation process can be found in the Supplemental Material Sections’ Equations (S1)–(S7).

From Equations (1) and (S1)–(S7), the Seidel coefficients can be expressed as the functions of *y*_1_, ${\bar{y}}_{1}$ and *c_i_* (*I* = 1, 2), *t_i_* (*i* = 1, 2, 3). Once the initial parameters are determined, we substitute them into the formulas to derive the expressions for each type of aberration. By solving these equations simultaneously, we obtain an initial coaxial structure that is free of aberrations.

**Figure 2:**
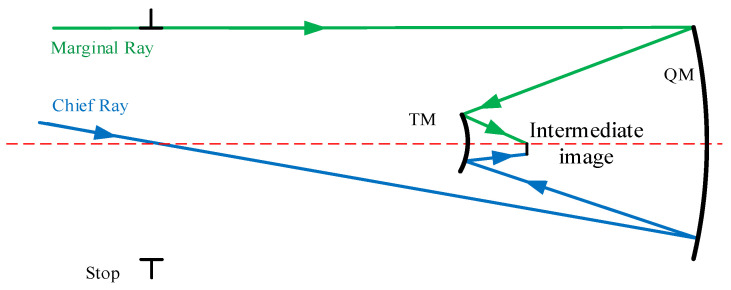
Raytracing of the initial coaxial TM and QM.

The design process of TM and QM is similar to that of PM and SM. The difference is that reverse ray tracing is used as shown in Figure 2, where a marginal ray and a chief ray are traced from the real exit pupil position.

Contrary to the conventional approach of constructing coaxial four-mirror structures, we directly apply off-axis modifications to the two-mirror coaxial structures to eliminate obscuration and observe the image quality at the intermediate image planes at that instant. This method is theoretically more efficient, which will be verified in Section 4. By simultaneously controlling the size, the global tilt angle, and the light beam numerical aperture (NA) of the intermediate image plane exactly the same in both off-axis two-mirror systems, we can directly obtain an initial off-axis structure where intermediate image planes are perfectly aligned. Subsequently, we will optimize the off-axis structure design, with a detailed exposition of the evaluation function design in the next subsection.

### 3.2. Simultaneous Multi-Configuration Design and Optimization

**Figure 3:**
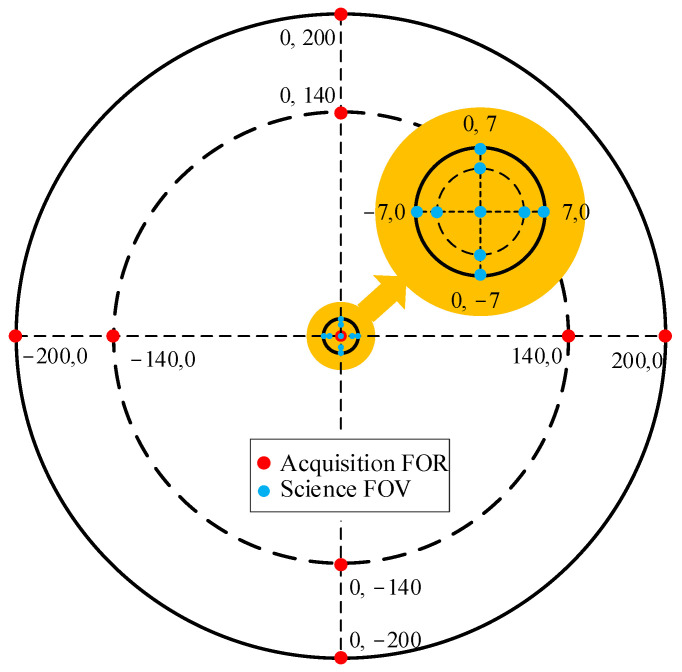
Multi-configuration field of view settings (units: μrad).

The detection of GW via space-based telescopes imposes stringent requirements on the wavefront errors within the science FOV, necessitating design precision on the order of λ/300. Within the acquisition FOR, the wavefront quality must also be considered to ensure adequate margins. Achieving both objectives within a single structure is challenging. This paper ingeniously adopts a simultaneous design approach, assigning different weights to the science FOV and acquisition FOR, and simultaneously optimizing the FOV in both ranges to guarantee high-quality beam transmission. As shown in Figure 3, Field sampling is conducted using a circular domain, which is advantageous as both the field of view and all the surfaces are symmetrical about the YOZ plane. In order to ensure uniform and effective field of view sampling, the center field of view, edge field of view, and 0.7 field of view are selected.

After setting the initial parameters according to the method in the previous subsection, we adopt the Damped Least Squares (DLS) algorithm to optimize the optical system. The merit function $L\left(\alpha \right)$ is defined as follows:

(2) $$\alpha^{*}=\underset{\alpha }{\arg\min}L\left(\alpha \right),$$

(3) $$L\left(\alpha \right)=\sum MF_{WFE}+\sum MF_{pupil}+MF_{constraints}.$$

where α represents the optimization variable, which includes the shape parameters of the mirrors and the distances between the mirrors in the optical system. $MF_{WFE}$ includes the wavefront error for the science FOV and acquisition FOR, $MF_{pupil}$ represents pupil aberration, and $MF_{constraints}$ signifies structural constraints.

For all the given field sampling points above, the expression of the wavefront error evaluation function is articulated as follows:

(4) $$MF_{WFE}=\sum_{k}\frac{\sum_{k}\left(w_{k}W_{k}^{2}\right)}{w_{k}}-{\left(\sum_{k}\frac{\sum_{k}w_{k}W_{k}}{w_{k}}\right)}^{2},$$

where $W_{k}$ is the optical path difference (OPD) at a given point in the pupil for each given field of view and $w_{k}$ is the corresponding weight. The OPD is the difference between the optical path length of the ray and the optical path length of the chief ray. And, the calculation is referenced back to the difference between the ray path length at the system exit pupil. For each field of view, the pupil sampling follows the Gaussian Quadrature weighted ring and arm sampling method, ensuring precise computation.

**Figure 4:**
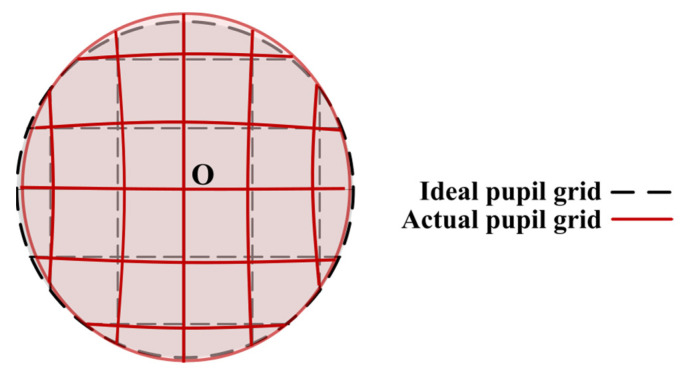
Pupil distortion caused by pupil aberration.

Within the spacecrafts, the entrance and exit pupils serve as apertures for receiving and transmitting light to subsequent optical systems. Therefore, the performance of light rays at the exit pupil of GW detection telescopes demands particular attention. As illustrated in Figure 4, the actual light ray sampling grid at the pupil exit will exhibit an offset compared to the ideal grid with no pupil aberrations. By employing a multi-configuration structure, the rays of the science FOV and acquisition FOR can be simultaneously traced and optimized for performance. In previous work [17], we have done a lot of derivations of pupil aberration, and its expression is more complex. We simplify the representation of pupil error to the representation of the chief rays, and each term of the pupillary function distortion is only related to the FOV vector, but not to the aperture vector.

The pupil aberration evaluation function $MF_{pupil}$, focusing on the distortion at the exit pupil. The optical aberration evaluation function focuses specifically on assessing the distortion at the exit pupil, thus highlighting its critical role in evaluating image quality. As a result, pupil aberration of the dominant light will appear as an offset in the center of the pupil as shown in Figure 4. In an ideal case, the chief rays must pass through the center of both the entrance pupil and the exit pupil without pupil aberrations. Conversely, if pupil aberration is present, the chief rays will deviate from the center of the entrance pupil. The greater the shift in the center of the telescope’s entrance pupil in different FOV, the greater the pupil aberration of the chief ray.

From the early stages of design, we have been particularly focused on the pupil aberrations of the telescope. The exit pupil is positioned 200 mm behind QM to ensure ample distance is available for the subsequent imaging system. Sampling is conducted at the intersection points of the chief rays across the full acquisition FOR at the exit pupil position, constraining the focal point to reduce the root mean square (RMS) of the chief ray spot diagram. The RMS radius of the chief rays was calculated as Equation (5).

(5) $$MF_{pupil}=R_{CRMS}=\sum_{i=1}^{N}\frac{\sqrt{{\left(x_{i}-\bar{x}\right)}^{2}+{\left(y_{i}-\bar{y}\right)}^{2}}}{N},$$

where *N* is the number of sampling points, $x_{i}$ denotes the *x*-coordinate of the *i*th sampling point on the pupil plane, $y_{i}$ denotes the *y*-coordinate of the *i*th sampling point on the pupil plane, and $\bar{x}$ and $\bar{y}$ represent the mean *x*-coordinates and *y*-coordinates of all sampling points, respectively.

The structural constraints evaluation function $MF_{contraints}$ consists of three parts. The first is fixed distance constraints, which include the entrance pupil to PM distance and the exit pupil to QM distance. The second is variable distance constraints, setting reasonable upper and lower limits, where the system’s total axial length should not exceed 550 mm, the vertical height not exceeding 300 mm, and the axial distance deviation between SM and the entrance pupil should not exceed ±5 mm to facilitate mechanical structure design. The third part is layout constraints, which are intended to prevent the beam from obstructing the mirror surface, requiring a minimum of 5 mm clearance from the top and bottom edges of the mirror surface.

## 4. Design Process and Results

### 4.1. Initial System Layout and Parameters

According to the design specifications and the aberration calculation method, the initial structure field of view is set to the acquisition FOR range, so $y_{1}$ = 110 mm, $u_{1}$ = 0, ${\bar{y}}_{1}$ = 0, ${\bar{u}}_{1}$ = 7 × 10^−6^ rad, and $t_{1}=-t_{2}=300$ mm. For the design process, Seidel coefficients are expected to be zero to achieve good image quality and $y_{3}$= 0 ought to be satisfied, thus the initial optical parameters of the system can be obtained.

**Figure 5:**
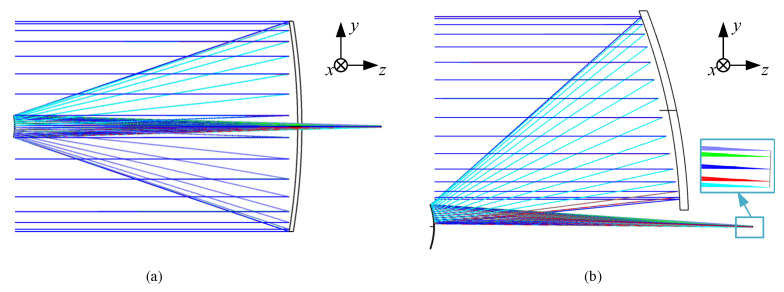
The initial layout of PM and SM: (**a**) coaxial layout after calculation; (**b**) off-axis layout.

Through aberration calculation, the Seidel coefficients of $S_{I}$~$S_{V}$ of this coaxial structure are all less than 1 × 10^−5^, which basically meets the aberration correction. The layout diagram of the coaxial two-mirror result obtained after implementing in the optical design software Ansys ZEMAX OpticStudio 2023 is shown in Figure 5a. By maintaining the system’s symmetry about the *x*-center field, namely the YOZ plane in Figure 5, perform elements decenter and tilt on this structure to eliminate central obscuration, resulting in an off-axis layout structure as shown in Figure 5b. The specific parameters of the off-axis initial structure are shown in Table S1: Coaxis parameters of PM and SM.

**Figure 6:**
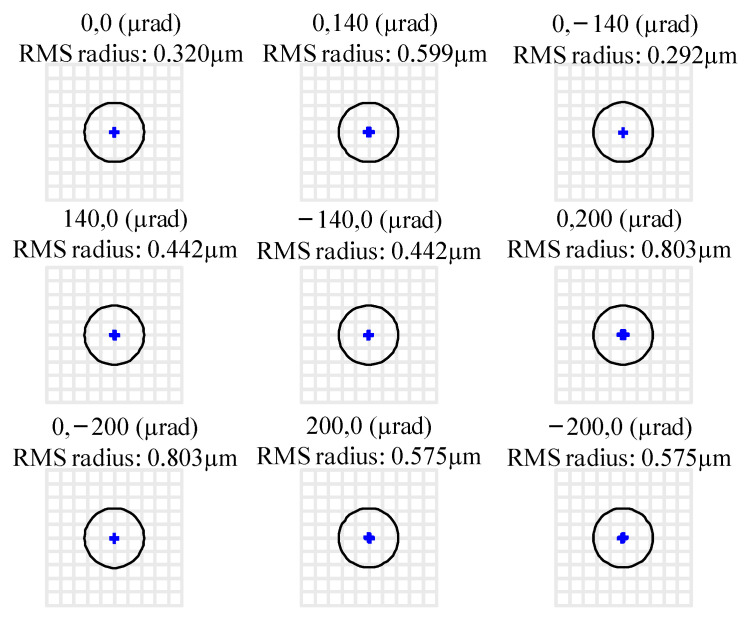
PM-SM initial structure spot diagram.

Compared with directly constructing a coaxial four-mirror structure and off-axis processing of PM and SM, we can effectively observe the imaging quality of the real intermediate image plane. The spot diagram performance of the image plane of the off-axis system in Figure 5b is shown in Figure 6, where the circle is the Airy disk with a radius of 22.06 µm. The imaging quality of the intermediate image plane after off-axis processing still reaches the diffraction limit. The image plane diameter is 1.57 mm, which can set a field stop here to control stray light.

**Figure 7:**
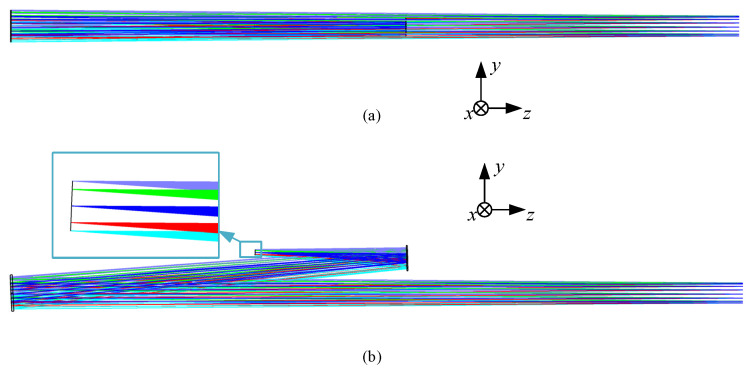
Initial off-axis system layout of TM and QM: (**a**) co-axial layout after calculation; (**b**) off-axis layout.

After calculation, the Seidel aberration coefficients $S_{I}$~$S_{V}$ of the initial coaxial structure are all less than 1 × 10^−6^, achieving aberration correction. The initial coaxial structure layout is shown in Figure 7a. Similarly, decenter and tilt TM, QM, and the intermediate image plane t eliminate obscuration, ensuring that the tilt angle at the intermediate image plane matches exactly with Figure 5b as well. The off-axis initial structural layout is obtained as shown in Figure 7b, and the structural parameters are shown in Table S2. Co-axis parameters of QM and TM.

**Figure 8:**
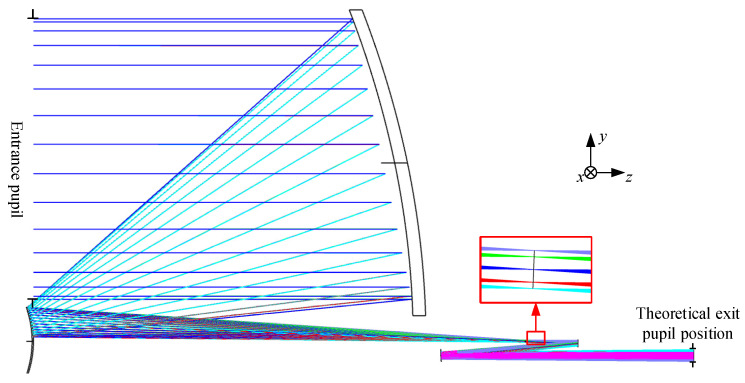
Initial off-axis four-mirror system layout.

Next, connect the off-axis two-mirror systems in Figure 5b and Figure 7b at the intermediate image plane and still set the entrance pupil 300 mm in front of PM. Figure 6 shows the initial structural layout of the four-mirror obtained by direct connection. The theoretical exit pupil position should be consistent with the stop in Figure 5b, which is located 200 mm behind the four mirrors. The connection at the center image plane is perfect, and the spot diagram performance here is shown in Figure 3. However, due to the existence of pupil aberration, as shown in Figure 8, there is an error between the theoretical exit pupil position and the actual exit pupil after direct connection. This is also a part that needs to be optimized in the subsequent design.

**Figure 9:**
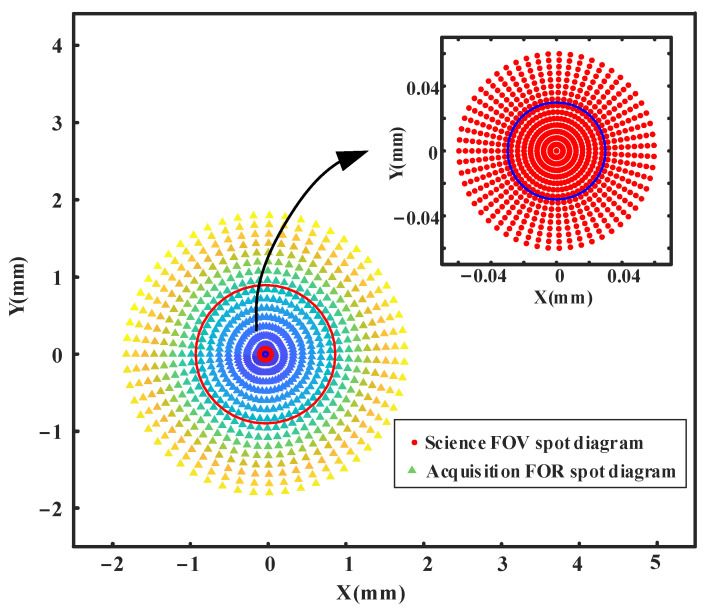
Chief ray spot diagram of Initial off-axis four-mirror system at the theoretical exit pupil position.

In order to evaluate the light performance of the theoretical exit pupil surface at this time, the chief rays of the science FOV and acquisition FOR were traced, and the spot diagrams of the chief rays are observed, as shown in Figure 9. Although there is a connection error at the theoretical pupil plane, the chief ray spot diagram after direct connection performs well, the spot shape is almost a perfect circle, and there is very little distortion. A reasonable initial structure can be obtained efficiently by this method.

Including the central science FOV, 880 sampling points are selected on the full circle FOR to calculate *R*_CRMS-F_ = 0.897 mm and *R*_CRMS-S_ = 0.030 mm according to Equation (5), which are represented by a red circle and blue circle in Figure 9, respectively.

### 4.2. Final Designed System Layout and Detailed Parameters

**Figure 10:**
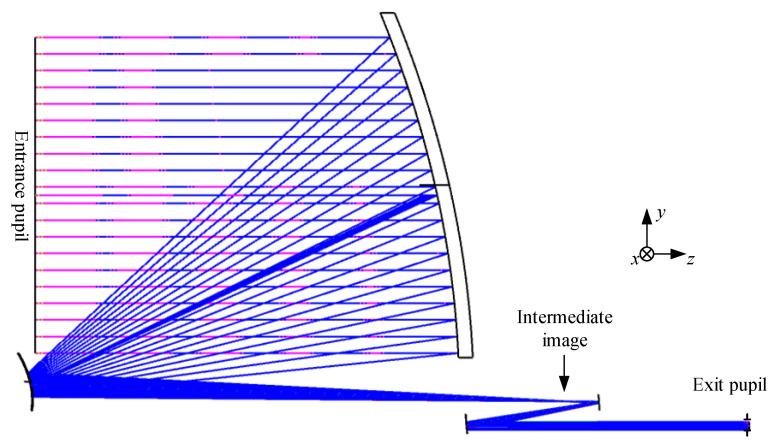
Final system layout of GW detection telescope.

The optimization process follows Equations (2)–(5), where we optimize the wavefront errors, pupil aberrations, and structural constraints under the multi-configuration of the science FOV and Acquisition FOR. By flexibly allocating weights to different fields of view, the evaluation function *L* (α) converges to the optimal solution. The final layout of the GW detection telescope system is illustrated in Figure 10. It features a 220 mm large-aperture parabolic PM, with SM and QM being even-order aspherical and TM being a standard spherical surface. Compared to previous similar designs [16,17,19], this updated configuration maintains high-precision wavefront transmission while utilizing only two even-order aspherical surfaces, thereby significantly reducing the complexity of alignment and manufacturing. The system contains an intermediate image plane with a total optical length of 400.18 mm, a length in the *y* direction of 275 mm, and a thickness in the *x* direction of 220 mm, which is the same as the entrance pupil diameter. This design layout is well-balanced and meets the predetermined requirements. The entrance pupil is positioned 300 mm in front of PM, while the actual exit pupil is located 200 mm behind QM, both aligned along the *z*-axis. The final design’s surface and spatial layout parameters are shown in Table 2. Both SM and QM utilize eighth-order even aspheric surfaces, with the specific coefficients detailed in Table 3.

**Figure 11:**
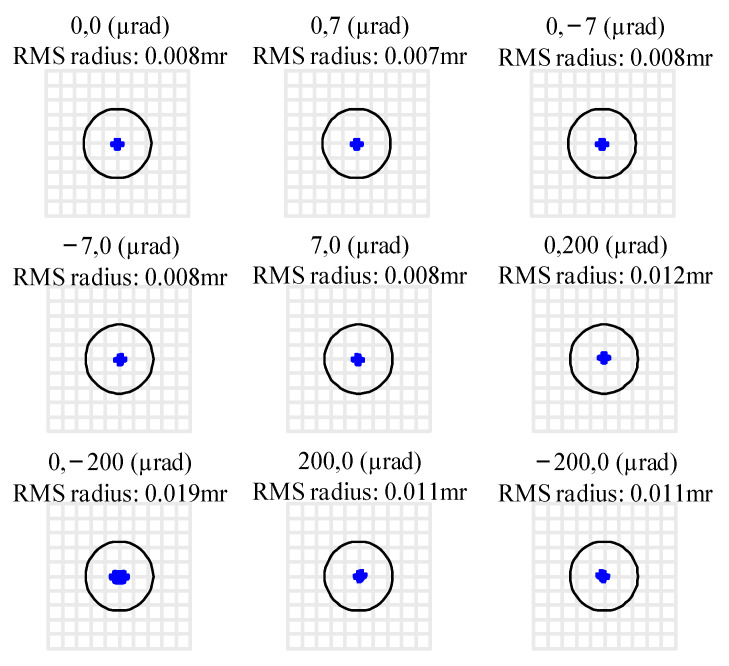
The spot diagram of the improved GW detection telescope system.

For afocal systems, the image-side spot diagram is expressed in degrees, and the Airy radius representing the diffraction limit is equally accurate and can be used directly as an evaluation criterion. Therefore, the spot diagram at the exit pupil of this design is illustrated in Figure 11, where the radius of the Airy disk is 0.239 mrad. It can be observed that the final system maintains excellent collimation across the entire science FOV with a maximum RMS radius of 0.007 mrad and 0.019 mrad in FOR. The root mean square (RMS) radius of all spots across FOV is significantly smaller than that of the Airy disk, with the primary components of residual aberrations being spherical and coma aberrations.

## 5. Performance Analysis of Final System

### 5.1. Wavefront Error RMS

**Figure 12:**
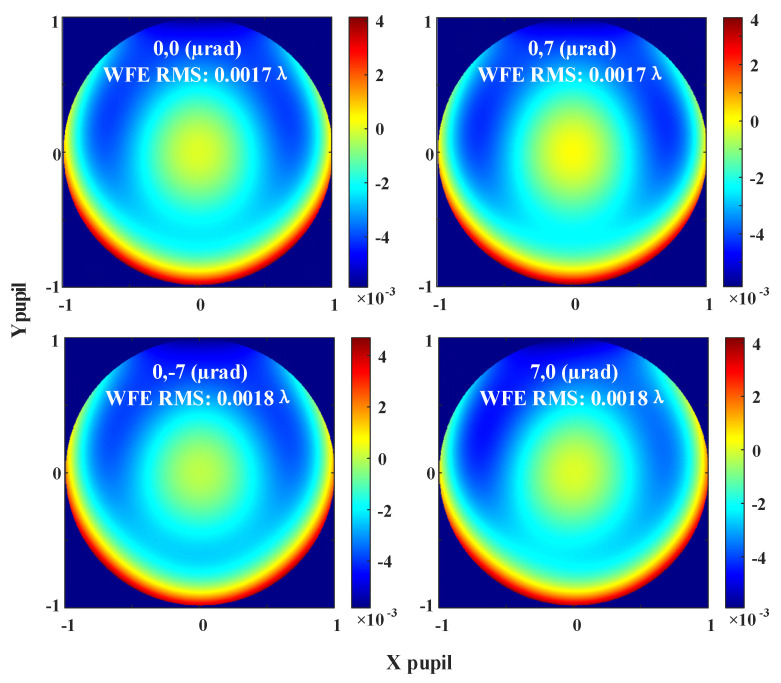
The wavefront error across the science FOV of the improved telescope system.

When evaluating the wavefront error of an afocal system like the GW detection telescope, two common methods are typically employed. One is to observe the wavefront change directly at the telescope exit pupil position, and the other is to observe the wavefront error of the focused image plane through an ideal lens. Since the ideal lens does not introduce additional aberrations, these two analyses are completely equivalent. We observe the wavefront performance directly at the exit pupil position, as shown in Figure 12. Since the performance of the whole science FOV is not much different, the center and edge of the pupil of the entire FOV are taken. And, the (−7 μrad, 0) field of view and the (7 μrad, 0) wavefront are symmetrical about the plane YOZ, the error performance is exactly the same, which is omitted in the figure. The system’s root mean square wavefront error is no greater than λ/500, ensuring the laser beam’s high wavefront quality after expansion and this result is much better than previous results of λ/300 [16].

### 5.2. The Chief Ray Diagram at the Exit Pupil Position

**Figure 13:**
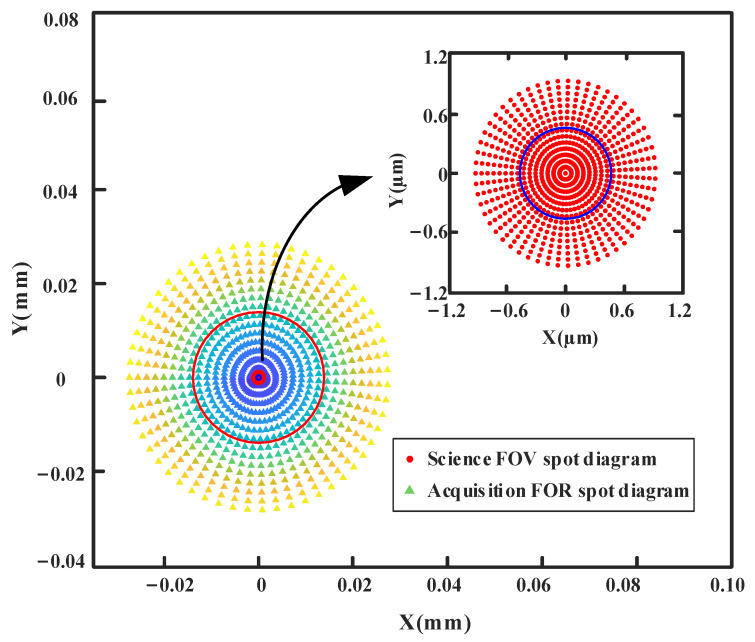
Chief ray spot diagrams at the exit pupil.

After controlling the morphology of the chief rays across the entire acquisition FOR at the pupil plane, the final structure achieves excellent design outcomes in controlling pupil plane aberrations. Through direct data link communication of MATLAB and Zemax, the chief rays of the science FOV and acquisition FOR were traced, and the spot diagrams of the chief rays on the exit pupil were observed, as shown in Figure 13.

Including the central science FOV, 880 sampling points on the full circle FOR are selected to calculate *R*_CRMS-F_ = 0.0139 mm (red circle in Figure 13) and *R*_CRMS-S_ = 0.464 μm (blue circle in Figure 13), which are 65 times smaller than those in initial structure. The chief ray RMS radii of this improved design telescope are much smaller than designs before, and the shape of the spot diagram is close to a perfect circle, indicating that the pupil aberration is well controlled successfully. If there is a higher demand for pupil aberration in the future, TM may be replaced with freeform surfaces to further correct pupil aberration. This result represents significant progress compared to previous results in the millimeter range [15,17,25].

### 5.3. TTL Noise Analysis

TTL noise is considered one of the most significant sources of noise aside from shot noise. Therefore, another design objective is to eliminate the TTL coupling in the transceivers caused by continuous jitter during GW measurements. After propagating over hundreds of thousands of kilometers, the wavefront at the distant end of the telescope can be approximated as a plane wave. By calculating the complex phase of the overlap integral, the difference in propagation path length between the tilted measurement beam and the Gaussian beam from the local optical bench can be determined [20,26].

(6) $$\arg\left(\int_{S}{E_{meas}E_{ref}}^{*}dr^{2}\right)=k\cdot s,$$

where the wavenumber *k* = 2*π*/*λ*. The change in OPL with angle provide quantitative index to describe the TTL coupling noise with units of length/angle.

In this study, more terms (the first 37 terms) of the Fringe Zernike polynomials are used to fit the wavefront error in measuring beam. The measuring beam with a wavefront residual Δ*W* can be expressed as:

(7) $$E_{meas}\left(x,y,z\right)=A_{meas}\exp\left\{-ik\left(x\sin\alpha +z\cos\alpha \right)+2\pi i\Delta W\right\},$$

where *α* is the angle of jitter.

**Figure 14:**
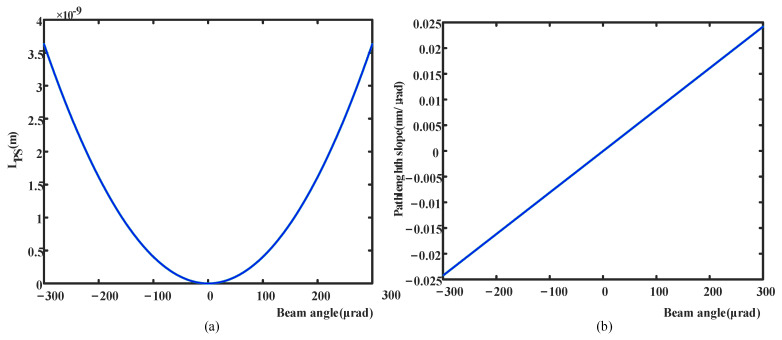
Analysis of TTL coupling noise. (**a**) The curve of LPS with beam angle; (**b**) TTL noise caused by wavefront error.

According to the science FOV of ±7 μrad and the magnification factor of 40×, we need to consider the TTL noise within ±280 μrad. In this paper, we extended the range to ±300 μrad. In this range, we continuously change the beam angle to observe the corresponding change in the longitudinal path length signal (LPS), and the simulation results are shown in Figure 14a. Within the entire ±200 μrad FOV, the TTL noise of this design is 0.015 nm/μrad approximately, satisfying noise budget, which is shown in Figure 14b. Considering spacecraft jitter, the maximum TTL noise within the ±300 μrad FOV is currently slightly less than 0.025 nm/rad, indicating the potential need for additional measures to suppress TTL at the edges of the FOV. Numerical simulations and experiments demonstrate that the use of imaging systems can mitigate beam wandering on photodetectors, thereby reducing TTL coupling. Another method to suppress TTL noise is to position the beam waist appropriately and utilize adequately sized detectors without causing clipping. Further work is required to achieve these designs for TTL noise suppression on the optical bench and to consider some trade-offs with the telescope design.

## 6. Additional Analysis of the Final System

### 6.1. Tolerance Analysis

We conducted verification of the tolerance performance of the telescope designed in this study to minimize progress risks and engineering costs. Utilizing the wavefront tolerance analysis feature in optical design software enables the assessment of the optical system’s sensitivity to manufacturing and assembly errors. Considering the installation of TM and QM on a movable platform, we can use the distance between them and the first two mirror surfaces as a compensator. Additionally, the back focal distance, i.e., the distance between QM and the exit pupil, is selected as another compensator. Those two compensation ranges are both set to ±3 mm. The wavefront error of the telescope is primarily caused by surface figure errors of mirrors and assembly calibration. Surface errors result from machining inaccuracies and assembly deformations, with errors in curvature radius being classified under surface errors and not subject to additional analysis. The detailed tolerance allocation is listed in Table 4, where the thickness represents the distance error of the current surface to the next surface. The surface irregularity error of the mirrors is simulated using the Zernike polynomial 2~22 terms (excluding the piston term).

**Figure 15:**
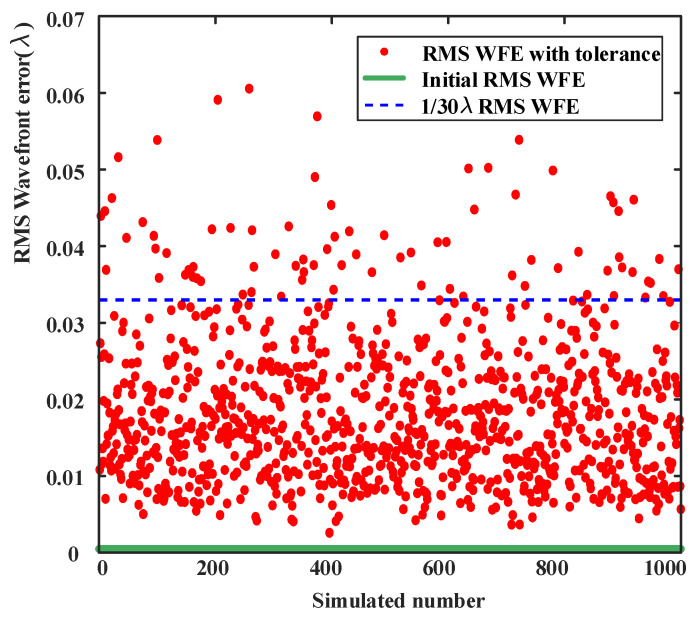
RMS WFE distribution of Monte Carlo 1000 trials.

According to the above settings, 1000 trails of Monte Carlo analysis were performed to predict the RMS wavefront error of the as-built system. Each parameter is perturbed with a random value within the tolerance range for each variable. The distribution of the 1000 simulation results is shown in Figure 15. Within the science FOV, most of the perturbation results are less than λ/30. Statistical analysis of the simulation data shows that the probability that the wavefront RMS error is less than 0.0333λ is 92%, which is completely acceptable and leaves room for other possible error sources. The detailed data are listed in Table 5.

### 6.2. Stray Light Analysis

Although a complete description of the stray light analysis is outside the scope of this paper, we also conducted a simple stray light analysis of this system and presented the result here. At the theoretical analysis level, only the scattered light caused by surface roughness and surface particles inside the optical system is considered. With the help of the commercial software ASAP 2019, the Harvey Shake scattering model is used to simulate all mirrors, which are coated with a thin film with a reflectivity of 0.99. In the established simulation model, there are no other baffles except the field baffle in front of the detector. We set the particle size range to 5 μm to 30 μm. The surface percentage coverage, that is, the ratio of the sum of the projected areas of all particles to the surface area when the incident wave illuminates the mirror perpendicularly, is set to 10^−3^. Then, the stray light received by the detector within the FOV of the telescope is numerically simulated. The cleanliness is CL200, and the surface roughness is 0.5 nm, which can meet the design requirements. It should be specially noted that the relevant specific analysis theories and data will be carried out and presented in subsequent work. The analysis results we obtained are close to the surface roughness requirements of the LISA project [27,28], but there are still difficulties in manufacturing. Due to the limitations of current manufacturing technology, the achievable roughness is closely related to the mirror design, so it is necessary to take some measures in other aspects to reduce manufacturing requirements in order to reduce cost and schedule risks.

### 6.3. Thermal Analysis

**Figure 16:**
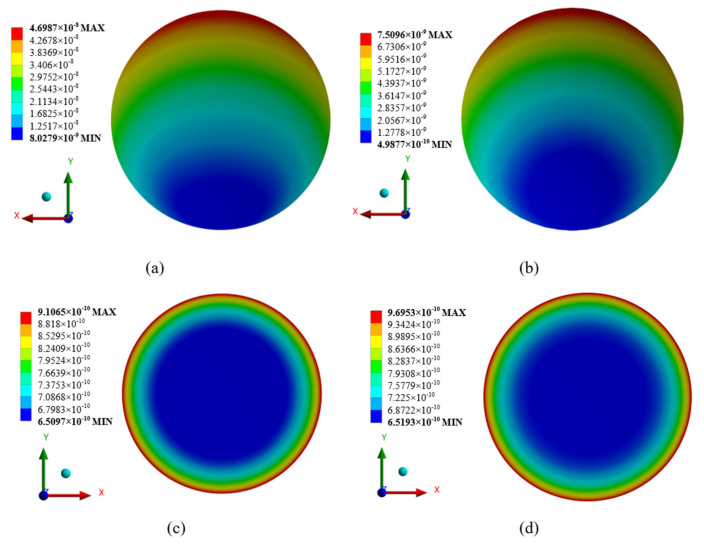
Displacement nephogram of mirrors: (**a**) PM; (**b**) SM; (**c**) TM; (**d**) QM.

As the design demonstration of optomechanical structure integration has not been completed yet, this paper does not conduct a complete discussion on thermal analysis and integration. The thermal analysis in this paper only focuses on the surface deformation of the mirror caused by environmental temperature changes. The mirror material is planned to use Zerodur with an extremely small linear expansion coefficient, and the support structure is planned to use invar with a matching linear expansion coefficient to eliminate the impact of thermal changes. The LISA-like project requires an orbital temperature stability on the order of 1 × 10^−5^ K [29]. Here, we have simulated and tested the deformations of PM and SM when the environmental temperature changes by 20 °C. This temperature range exceeds the temperature change of the on-orbit working environment. The deformation nephograms of the four mirrors are shown in Figure 16. The RMS surface errors of PM, SM, TM, and QM caused by surface deformation are 1.01 × 10^−8^ m, 1.94 × 10^−9^ m, 6.65 × 10^−11^ m, and 9.03 × 10^−11^ m, respectively. This result is similar to the tolerance sensitivity result, where the deformations of PM and SM are much greater than those of TM and QM. The results also show that even if the ambient temperature changes by 20 degrees, the degradation of the surface error is still lower than the manufacturing tolerance of 10 nm, and the wavefront quality is acceptable to the design requirement of λ/30 for the telescope subsystem. In actual opto-mechanical structure integration and on-orbit operation, more factors will be comprehensively considered for the thermal effect of the system, and the use of new materials is expected to further reduce the impact of thermal effects.

## 7. Conclusions

In this paper, we make improvements on the previous design work [16,17] regarding the telescope system for GW detection, in which the mirror space position is reasonable, for example, the entrance pupil is 300 mm in front of PM, and the exit pupil is 200 mm behind QM. In terms of construction method, starting from the Seidel aberration theory, the aberration calculations of PM and SM, TM, and QM are, respectively carried out to construct the initial structure, and the system is first off-axis adjusted and then directly integrated. This approach ensures a structurally rational initial configuration with high image quality, enhancing design efficiency. The initial structure obtained in this way can ensure a reasonable structure, good image quality, and improve the design efficiency. When optimizing the design, simultaneously optimize the acquisition FOR, science FOV, and control pupil error to meet the requirements of low distortion and high-quality wavefront transmission. The final telescope design features a reasonable configuration, provides sufficient space for subsequent mechanical system design and imaging systems, and is capable of achieving an ultra-low RMS wavefront error of λ/500. In addition, the tolerance analysis of this system shows that the system has good manufacturability. Under normal optical processing and cleaning levels, the design has relatively high performance and ultra-low backscattering, which can well meet the scientific requirements of GW detection. Therefore, this design can meet the GW detection mission, providing valuable design methods and strategies for GW detection telescopes. Future work can be continued around the final system in this paper, focusing on aspects such as mirror support, stray light suppression structures, integrated opto-mechanical system, lightweighting, stability, etc.

## Figures and Tables

**Table 1:** Key technology requirements of the telescope.

Parameter	Specification
Wavelength	1.064 μm
Acquisition FOR	±200 μrad
Science FOV	±7 μrad
Entrance pupil diameter	220 mm
Afocal magnification	40×
Entrance pupil position	300 mm before PM
Exit pupil position	200 mm behind QM
Wavefront RMS (design residual)	≤λ/300
TTL noise	≤0.025 nm/µrad

**Table 2:** Off-axis structure parameters of improved design.

Surface	Radius/mm	Distance/mm	Conic	Y Decenter/mm	X Tilt/°
Stop	-	300	-	0	0
PM	−651.79	−301.49	−1	−143.01	0
SM	−52.21	400.18	−1.684	0	0.04
TM	636.56	−93.69	0	−1.29	−4.15
QM	232.92	200.00	−3.377	−16.27	−4.65
XP	-	-	-	−1.518	4.62

**Table 3:** Detailed even-order aspheric coefficients of SM and QM.

Surface	4-th Order	6-th Order	8-th Order
SM	−3.3700 × 10^−7^	5.9477 × 10^−11^	−1.0259 × 10^−14^
QM	−1.2660 × 10^−7^	4.6580 × 10^−9^	−6.1568 × 10^−11^

**Table 4:** Tolerance setting of sensitivity analysis.

Surface	Radius	Distance	Decenter in *x*	Decenter in *y*	Tilt in *x*	Tilt in *y*	Irregularity
Stop	-	±10 μm	-	-	-	-	-
PM	±10 μm	±10 μm	±0.5 μm	±0.5 μm	±0.001°	±0.001°	1/60*λ*
SM	±10 μm	±3 mm	±0.5 μm	±0.5 μm	±0.001°	±0.001°	1/60*λ*
TM	±50 μm	±10 μm	±1 μm	±1 μm	±0.001°	±0.001°	1/60*λ*
QM	±50 μm	±3 mm	±1 μm	±1 μm	±0.001°	±0.001°	1/60*λ*

**Table 5:** Monte Carlo tolerance analysis probability results (1000 Trials).

Criterion	Worst	92%	90%	80%	50%	20%	10%	2%
RMS WFE/*λ*	0.0635	0.0333	0.0316	0.0250	0.0164	0.0102	0.0081	0.0056

## Data Availability

All data that support the findings of this study are included within the article (and any Supplementary Files).

## Supplementary Materials

The following supporting information can be downloaded at: https://www.mdpi.com/article/10.3390/s24227309/s1, Equations (S1)–(S7); Table S1: Coaxis parameters of PM and SM; Table S2: Coaxis parameters of QM and TM.
