# Supplementary material for: High-Performance Telescope System Design for Space-Based Gravitational Waves Detection
Source: Sensors (Basel). 2024 Nov 15;24(22):7309. doi: 10.3390/s24227309 (PMC11598358; doi:10.3390/s24227309)
Supplement: Supplementary file 1 [file sensors-24-07309-s001.zip › sensors-3285061-supplementary.pdf]

sensors

MDPI

Article

High-Performance Telescope System Design for Space-Based Gravitational Waves Detection

Huiru Ji [1], Lujia Zhao [1,*], Zichao Fan [1], Rundong Fan [2], Jiamin Cao [1], Yan Mo [1], Hao Tan [1], Zhiyu Jiang [1] and Donglin Ma [1,2,3,*]

[1] MOE Key Laboratory of Fundamental Physical Quantities Measurement and Hubei Key Laboratory of Gravitation and Quantum Physics, PGMF and School of Physics, Huazhong University of Science and Technology, Wuhan 430074, China; huiruji@hust.edu.cn (H.J.); fanzichao@hust.edu.cn (Z.F.); caojiamin@hust.edu.cn (J.C.); ammo0925@hust.edu.cn (Y.M.); tanhao960410@hust.edu.cn (H.T.); jiangzy@hust.edu.cn (Z.J.)
[2] School of Optical and Electronic Information and Wuhan National Laboratory of Optoelectronics, Huazhong University of Science and Technology, Wuhan 430074, China; frd0823@hust.edu.cn
[3] Shenzhen Huazhong University of Science and Technology, Shenzhen 518057, China
* Correspondence: d202080060@hust.edu.cn (L.Z.); madonglin@hust.edu.cn (D.M.)

1. Formula Derivation

For marginal ray, the relationship between the ray height and the ray slope on different mirrors can be determined by the paraxial ray tracing formulas, which are expressed as

$$\begin{cases} n_i' u_i' = n_i u_i - y_i \phi_i \\ y_{i+1} = y_i + u_i' t_i' \\ t_i' = t_{i+1} \\ u_i' = u_{i+1} \end{cases}, \tag{S1}$$

where i ($i = 1,2$) represents PM, SM respectively, n_i and n_i' represent the transmission media refractive index, u_i is the paraxial ray's incident slope with respect to the optical axis, and u_i' is the corresponding exit slope. ϕ_i is the optical power of mirrors, y_i represent the heights of the marginal ray on PM and SM, and t_3 represents the distance between SM and the intermediate image plane.

The ray-tracing calculation method of the chief ray is similar to that of the marginal ray. The extension line of the incident ray passes through the center of the entrance pupil and the ray tracing follows

$$\begin{cases} n_i' \bar{u}_i' = n_i \bar{u}_i - \bar{y}_i \phi_i \\ \bar{y}_{i+1} = \bar{y}_i + \bar{u}_i' t_i' \\ t_i' = t_{i+1} \\ \bar{u}_i' = \bar{u}_{i+1} \end{cases}, \tag{S2}$$

where $\bar{y}_i$ represents the height of the chief ray, and $\bar{u}_i$ is the slope of the chief ray.

According to the Seidel aberration theory, in a rotationally symmetric spherical optical system, five monochromatic aberrations, including the spherical aberration (S_{I}^s), coma (S_{II}^s), astigmatism (S_{III}^s), Petzval curvature of field (S_{IV}^s), and distortion (S_{V}^s), can be expressed by the structural parameters of the system as

$$\begin{cases} S_{\mathrm{I}}^{s} = -\sum A^{2}\cdot y\cdot \Delta(\frac{u}{n}) \\ S_{\mathrm{II}}^{s} = -\sum A\overline{A}\cdot y\cdot \Delta(\frac{u}{n}) \\ S_{\mathrm{III}}^{s} = -\sum \overline{A}^{2}\cdot y\cdot \Delta(\frac{u}{n}) \\ S_{\mathrm{IV}}^{s} = -\sum L^{2}\cdot c\cdot \Delta(\frac{1}{n}) \\ S_{\mathrm{V}}^{s} = -\sum \left\{ \frac{\overline{A}^{3}}{A}\cdot y\cdot \Delta(\frac{u}{n}) + \frac{\overline{A}}{A}\cdot L^{2}\cdot c\cdot \Delta(\frac{1}{n}) \right\} \end{cases}, \quad (S3)$$

where A is the refraction invariant of the marginal ray, $\overline{A}$ is the refraction invariant of chief ray, c is surface curvature, and L represents the Lagrange invariant of the system. The refraction invariants on the current surface and Lagrange invariants can be obtained by Equation S4:

$$\begin{cases} A = n(yc + u) \\ \overline{A} = n(\overline{y}c + \overline{u}) \\ L = n\overline{u}y - nu\overline{y} \end{cases}. \quad (S4)$$

For conic surfaces, the introduction of aspherical sagittal height on the surface will introduce corresponding aspherical aberration. The sag of conic surface is defined by the following equation:

$$z(x, y) = \frac{cr^{2}}{1 + \sqrt{1 - (1+\kappa)c^{2}r^{2}}}, \quad (S5)$$

where κ is the conic constant, and $r^{2} = x^{2} + y^{2}$. Therefore, the change of the vector height of the aspheric surface can be expressed as:

$$\Delta z = \frac{1}{8}\kappa c^{3} r^{4}. \quad (S6)$$

When the conic surface is not at the pupil, additional spherical aberration ($\delta S_{\mathrm{I}}^{a}$), coma ($\delta S_{\mathrm{II}}^{a}$), astigmatism ($\delta S_{\mathrm{III}}^{a}$) and distortion ($\delta S_{\mathrm{V}}^{a}$) of each mirror will be introduced, and the expressions are as follows:

$$\begin{cases} \delta S_{\mathrm{I}}^{a} = \sum \kappa c^{3} y^{4} \Delta(n) \\ \delta S_{\mathrm{II}}^{a} = \frac{\overline{y}}{y}\delta S_{\mathrm{I}}^{a} \\ \delta S_{\mathrm{III}}^{a} = \left(\frac{\overline{y}}{y}\right)^{2} \delta S_{\mathrm{I}}^{a} \\ \delta S_{\mathrm{V}}^{a} = \left(\frac{\overline{y}}{y}\right)^{3} \delta S_{\mathrm{I}}^{a} \end{cases}, \quad (S7)$$

2. Tables

Table S1. Coaxis parameters of PM and SM.

Surface	Radius/mm	Distance/mm	Conic	Y Decenter/mm	X Tilt/°
Stop	-	300.00	-	0	0
PM	−669.63	−300.00	−1.00	−143.00	0
SM	−76.48	388.58	−1.44	0	0
IMAGE	-	-	-	0	2.19

Table S2. Coaxis parameters of QM and TM.

Surface	Radius/mm	Distance/mm	Conic	Y Decenter/mm	X Tilt/°
STOP	-	−200	-	0	−1.06
QM	390.93	108.61	−6.34	10	0
TM	−158.98	−41.68	0.98	−4.4	0
IMAGE	-	-	-	5.6	2.19
